# Disparities and Trends in Indoor Exposure to Secondhand Smoke among U.S. Adolescents: 2000-2009

**DOI:** 10.1371/journal.pone.0083058

**Published:** 2013-12-17

**Authors:** Israel T. Agaku, Constantine I. Vardavas

**Affiliations:** Center for Global Tobacco Control, Department of Social and Behavioral Sciences, Harvard School of Public Health, Boston, Massachusetts, United States of America; University of Texas at Tyler, United States of America

## Abstract

**Introduction:**

Secondhand smoke (SHS) exposure causes disease and death among nonsmokers. With a plethora of smoke-free legislation implemented and a steady decrease in cigarette consumption noted over the past decade in the U.S., this study assessed trends in indoor SHS exposure among U.S. adolescents in grades 6–12 during 2000–2009.

**Methods:**

Data were obtained from the 2000–2009 National Youth Tobacco Survey – a national survey of U.S. middle and high school students. SHS exposure within an indoor area within the past seven days was self-reported. Trends in indoor SHS exposure during 2000–2009 were assessed overall and by socio-demographic characteristics, using the Wald's test in a binary logistic regression. Within-group comparisons were performed using chi-squared statistics (*p*<0.05).

**Results:**

The proportion of U.S. middle and high school students who were exposed to indoor SHS declined from 65.5% in 2000 to 40.5% in 2009 (*p*<0.05 for linear trend). Significant declines were also observed across all population subgroups. Between 2000 and 2009, prevalence of indoor SHS exposure declined significantly among both middle (58.5% to 34.3%) and high school (71.5% to 45.4%) students. Prevalence of indoor SHS exposure was significantly higher among girls (44.0% in 2009) compared to boys (37.2% in 2009) during each survey year. Similarly, prevalence of indoor SHS exposure during 2000–2009 was highest among non-Hispanic whites (44.2% in 2009) and lowest among non-Hispanic Asians (30.2% in 2009). During each survey year, prevalence was highest among the oldest age group (≥18 years) and lowest among the youngest (9–11 years). Also, prevalence was significantly higher among current cigarette smokers (83.8% in 2009) compared to nonsmokers (34.0% in 2009).

**Conclusion:**

Significant declines in indoor SHS exposure among U.S. middle and high school students occurred during 2000–2009. While the results are encouraging, additional efforts are needed to further reduce youth indoor SHS exposure.

## Introduction

Exposure to secondhand smoke (SHS) causes death and disease in both non-smoking adults and children, to which no safe level of exposure exists.[1] Each year, an approximate 46,000 heart disease deaths and 3,400 lung cancer deaths among non-smoking adults in the United States are attributable to SHS.[1] SHS exposure has also been associated with cancer, heart disease, asthma, lower respiratory tract illness, as well as a plethora of other adverse outcomes including neurologic disorders and impaired cognitive abilities in children.[1]–[3]

The scientific evidence indicates that there is no risk-free level of exposure to SHS. Eliminating smoking in indoor spaces fully protects nonsmokers from exposure to SHS. More so, comprehensive smoke-free policies which prohibit indoor smoking in all indoor public areas can change social norms towards smoking.[1], [4] Because of the changing social environment towards SHS exposure, the proportion of U.S. smoke-free households has increased in recent times.[2] In addition, over the past decade, there has been a proliferation of local and state policies prohibiting smoking in private and government workplaces, malls, enclosed arenas as well as hospitality venues such as bars, and restaurants.[5]–[8] However, in several states, adolescents and youths are still susceptible to SHS exposure in several indoor areas, including common areas of government and private multi-unit housing, living areas of residences, and other private areas.[6] Despite these developments, very little data is available on temporal trends in indoor SHS exposure among U.S. adolescents and how such exposure may differ by socio-demographic characteristics. Therefore, the aim of this study was to assess overall and subpopulation trends in indoor SHS exposure among U.S. middle and high school students between 2000 and 2009, using data from the National Youth Tobacco Survey (NYTS).

## Methods

### Data Sources

The NYTS is a repeated, biennial, self-administered cross-sectional survey that collects information on key tobacco-related measures from middle school (grades 6–8) and high school (grades 9–12) students in a classroom setting. The sampling frame consists of all public schools, Catholic schools, and other private school students enrolled in grades 6 to 12 in the 50 states and the District of Columbia. Alternative schools, special education schools, Department of Defense-operated schools, vocational schools, and students enrolled in regular schools unable to complete the questionnaire without special assistance are excluded.

The number of students who completed each NYTS survey wave (*n*) and overall response rates (%) between 2000 and 2009 were as follows: 2000 (*n* = 35,828; 84.1%), 2002 (*n* = 26,149; 74.2%), 2004 (*n* = 27,933; 82.0%), 2006 (*n* = 27,038; 80.2%) and 2009 (*n* = 22,679; 84.8%).

### Measures

#### Indoor SHS exposure

Self-reported indoor SHS exposure was assessed using the question “During the past 7 days, on how many days were you in the same room with someone who was smoking cigarettes?” Numeric responses ranged from “0” to “7”. Responses other than “0” were categorized as being exposed to SHS in an indoor area.

#### Current Cigarette Smoking

Cigarette smoking status was assessed using the question, “During the past 30 days, on how many days did you smoke cigarettes?” Categorical response options included the following: “0 days,” “1 or 2 days,” “3 to 5 days,” “6 to 9 days,” “10 to 19 days,” “20 to 29 days,” and “all 30 days”. Current smokers were respondents who indicated a response other than “0 days”.

#### Socio-demographic Characteristics

Socio-demographic characteristics that were assessed included: sex (boy or girl), age (9–11, 12–14, 15–17, or ≥18 years), race/ethnicity (non-Hispanic white, non-Hispanic black, non-Hispanic Asian or Hispanic), and school level (middle or high).

### Data Analysis

Linear trends from 2000 to 2009 were assessed using the Wald's test in a binary logistic regression model. For the regression analysis, orthogonal polynomials were developed to account for variations in time between survey years, and results were adjusted for current smoking status, age, sex, race/ethnicity and school level to control for any differences in population composition during the study period. Within-group comparisons were performed using chi-squared statistics. The direction of trends in indoor SHS exposure during 2000–2009 was assessed using estimates of relative percent change. All statistical tests were two-tailed and the level of statistical significance was set at *p*<0.05. Data were weighted to account for the complex survey design, and analyzed with Stata version 11 (StataCorp. 2009: College Station, TX).

## Results

**Figure 1:**
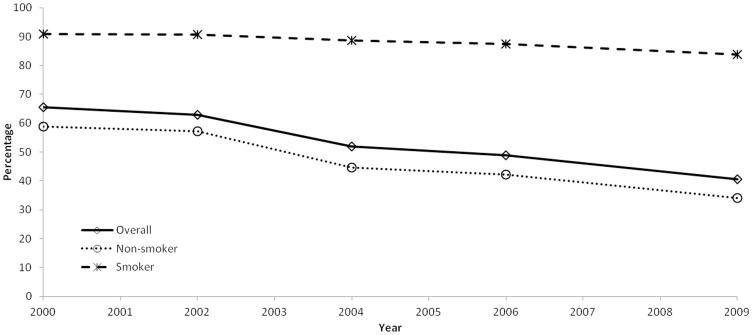
Proportion of middle and high school students who reported being exposed to indoor secondhand smoke within the past 7 days, overall and by current cigarette smoking status^*^, National Youth Tobacco Survey, United States, 2000–2009^†^. ^*^Current cigarette smokers were students who reported that they had smoked cigarettes on at least one day within the past 30 days preceding the survey. ^†^Significant declines in indoor exposure to secondhand smoke were observed overall (65.5% to 40.5%), as well as among current cigarette smokers (80.8% to 83.8%) and non-smokers (58.9% to 34.0%) (*p*<0.05 for all linear trends).

Significant declines in the prevalence of indoor SHS exposure between 2000 and 2009 were noted overall and across all sub-population groups of U.S. adolescents. Overall prevalence of indoor SHS exposure declined from 65.5% in 2000 to 40.5% in 2009 (*p*<0.05 for linear trend; **Table 1**, **Figure 1**). Between 2000 and 2009, significant downward trends were noted among both non-smokers (58.9% to 34.0%) and smokers (90.8% to 83.8%). Prevalence of indoor SHS exposure was significantly higher among current smokers compared to non-smokers during each survey year between 2000 and 2009.

**Table 1:** Proportion of middle and high school students who reported being exposed to indoor secondhand smoke within the past 7 days, National Youth Tobacco Survey, United States, 2000–2009.

Characteristics	2000% (95% CI) *n* = 35,828	2002% (95% CI) *n* = 26,149	2004% (95% CI) *n* = 27,933	2006% (95% CI) *n* = 27,083	2009% (95% CI) *n* = 22,679	2000–2009 Relative percentage Change
**Overall**	65.5 (63.7–67.4)	62.8 (61.0–64.7)	51.9 (49.6–54.1)	48.8 (46.5–51.1)	40.5 (38.2–42.8)	–61.7†
						
**Current smoking status** *						
Non-smoker	58.9 (57.1–60.8)	57.2 (55.4–59.0)	44.7 (42.9–46.5)	42.2 (40.2–44.3)	34.0 (31.8–36.1)	−73.2†
Smoker	90.8 (89.6–91.9)	90.6 (89.3–91.8)	88.7 (87.2–90.2)	87.5 (85.9–89.1)	83.8 (80.2–87.5)	−8.4†
**Age, years**						
9–11	51.3 (47.1–55.5)	51.6 (46.8–56.3)	36.7 (32.8–40.5)	33.7 (29.3–38.1)	27.9 (24.5–31.3)	−83.9†
12–14	59.8 (57.7–62.0)	57.5 (55.4–59.7)	45.7 (43.3–48.2)	42.5 (40.1–45.0)	34.8 (32.4–37.3)	−71.8†
15–17	71.2 (69.2–73.2)	67.4 (65.3–69.5)	58.0 (55.4–60.5)	55.2 (52.3–58.1)	45.8 (42.0–49.6)	−55.5†
≥18	76.3 (73.8–78.7)	74.7 (71.8–77.6)	64.5 (60.4–68.7)	61.5 (57.8–65.3)	52.9 (48.5–57.4)	−44.2†
**Gender**						
Girl	67.7 (65.8–69.6)	64.9 (63.0–66.9)	55.1 (52.6–57.7)	52.2 (49.6–54.8)	44.0 (41.5–46.6)	−53.9†
Boy	63.3 (61.3–65.4)	60.8 (58.7–62.8)	48.5 (46.3–50.6)	45.3 (43.1–47.5)	37.2 (34.0–40.3)	–70.2†
**Race/ethnicity**						
White, non-Hispanic	69.5 (67.4–71.6)	67.1 (64.8–69.3)	56.1 (53.2–59.0)	53.3 (50.5–56.2)	44.2 (41.4–47.0)	−57.2†
Black, non-Hispanic	60.0 (58.0–62.1)	56.5 (54.0–59.0)	43.8 (41.6–46.0)	39.8 (36.9–42.7)	31.6 (26.8–36.4)	−89.9†
Asian, non-Hispanic	53.4 (49.7–57.0)	48.2 (43.3–53.2)	35.2 (31.8–38.6)	31.7 (28.8–34.6)	30.2 (24.9–35.4)	−76.8†
Hispanic	54.9 (51.4–58.5)	53.2 (50.5–56.0)	44.7 (42.2–47.1)	41.9 (39.0–44.9)	38.9 (35.8–42.0)	−41.1†
**School level**						
Middle school (grades 6−8)	58.5 (56.3–60.7)	56.6 (54.3–59.0)	44.4 (41.9–46.9)	41.2 (38.4–44.0)	34.3 (31.8–36.8)	−70.6†
High school (grades 9–11)	71.5 (69.5–73.5)	67.9 (66.0–69.9)	58.1 (55.5–60.8)	55.2 (52.5–57.9)	45.4 (41.3–49.4)	−57.5†

***Note***: All percentages were weighted to adjust for differential non-response and selection; *n* = unweighted samples; *CI* = Confidence Interval.

* Current cigarette smokers were students who reported that they had smoked cigarettes on at least one day within the past 30 days preceding the survey.

† Statistically significant linear trend (*p* <0.05).

Significant declines were also noted across all age and racial/ethnic groups between 2000 and 2009. During each survey year, prevalence of indoor SHS exposure was highest among the oldest age group (i.e., students aged ≥18 years; 52.9% in 2009) and lowest among the youngest (i.e., students aged 9–11 years; 27.9% in 2009). Also, prevalence of indoor SHS exposure was highest among non-Hispanic whites (44.2% in 2009) and lowest among non-Hispanic Asians (30.2% in 2009) during 2000–2009. It is encouraging to note that while 60% of non-Hispanic blacks reported exposure to indoor SHS in 2000, the percentage reduced to 31.6% in 2009 (*p*<0.05 for linear trend). By sex, significant declines during 2000–2009 were observed among both girls (67.7% to 44.0%) and boys (63.3% to 37.2%). However, girls reported significantly higher exposure to SHS during each survey year compared to boys. By school level, prevalence of indoor SHS exposure declined significantly during 2000 and 2009, among both middle school (58.5% to 34.3%) and high school (71.5% to 45.4%) students. However, prevalence of indoor SHS exposure was significantly higher among high school students compared to middle school students during 2000–2009 (**Table 1**).

## Discussion

This study indicated that the proportion of U.S. middle and high school students who were exposed to indoor SHS declined from 65.5% in 2000 to 40.5% in 2009. Significant declines in indoor SHS exposure occurred across all population subgroups by age, gender, race/ethnicity, school level and smoking status. Despite these significant decreases, significant disparities in the extent of indoor SHS exposure were noted between different subpopulations. For example, the prevalence of indoor SHS exposure was significantly higher among current smokers compared to non-smokers; among non-Hispanic Whites compared to other groups such as non-Hispanic Asians; and among girls compared to boys. Also, prevalence was highest among the oldest age group and lowest among the youngest.

The higher prevalence of indoor SHS exposure among current smokers may be due to the fact that adolescents and youths who smoke are more likely to have peers, parents or siblings who are also current smokers, and less likely to have 100% smoke-free home rules.[9]–[11] Adolescent smokers may thus potentially be exposed to tobacco smoke not only from their active smoking, but also passively from proximal contacts, including smoking household members and peers. This underscores the need for enhanced and sustained interventions such as barrier-free access to clinical smoking cessation programs, and educational campaigns about the dangers of SHS exposure, especially targeted towards population subgroups with high smoking prevalence such as individuals of low socio-economic or educational status and those with a physical disability.[12] In addition, school tobacco-free policies which prohibit tobacco use by students, staff and visitors on all school grounds may also help in reducing adolescent exposure to indoor SHS at school.

The lower prevalence of indoor SHS exposure among non-Hispanic Asian students may be due to the relatively low smoking prevalence among both non-Hispanic Asian adults and youths,[12], [13] which may suggest lower likelihood of indoor SHS exposure in such households. The lower prevalence of indoor SHS exposure among students aged 9–11 compared to older students may be due to the relatively lower smoking prevalence among this younger group,[13] coupled with the fact that parents or older siblings who smoke may be less inclined to do so indoors in the presence of younger children. The higher prevalence of indoor SHS exposure among high school students when compared to middle school students may be related to susceptibility to social conformity and group identity at school and at social events, as well as the higher smoking prevalence among high school students.[13]

Despite the disparities in exposure to SHS among U.S. adolescents, the overall significant decline in indoor SHS exposure among U.S. middle and high school students during 2000–2009 is a major public health achievement and may be attributable to the proliferation of local and state comprehensive smoke-free laws prohibiting smoking in all indoor areas of private workplaces, restaurants, and bars, with no exceptions.[7], [8] Changing social norms towards exposure to secondhand smoke among non-smokers have resulted in a plethora of comprehensive smoke-free laws across the United States in recent years. From having no U.S. state with a comprehensive smoke-free law in 2000, 26 U.S. states and the District of Columbia had implemented such laws in 2013.[6] Such comprehensive smoke-free laws have been shown to benefit public health, and may have contributed to declines in SHS-associated conditions such as middle-ear infections, sudden infant death syndrome, and heart disease.[2], [14], [15] More so, in addition to achieving their primary objective of protecting nonsmokers from involuntary SHS exposure, such comprehensive smoke-free laws also denormalize smoking and may motivate smokers to quit.

While significant advances have been made in reducing SHS exposure in private workplaces, restaurants, and bars in the United States, relatively few regulatory entities have instituted restrictions on smoking in personal living areas, a route of exposure that may play a significant role in adolescents' exposure to indoor SHS.[16] Multi-unit housing residents — even those with strictly enforced non-smoking house rules — may still be susceptible to involuntary SHS exposure due to drift of tobacco smoke from adjacent apartments or common areas.[17] Indeed, a recent national study indicated that over half of multiunit housing residents with smoke-free home rules have experienced SHS infiltration.[18] Hence, the U.S. Federal Agency for Housing and Urban Development has recommended smoke-free policies in public housing units.[19] Private landlords, public housing authorities and other affordable housing owners may also implement non-smoking rules as part of the lease binding on all residents, rather than leaving it to individual residents' volition or discretion to implement their own non-smoking house rules. In addition, non-smokers, particularly those with severe respiratory or cardiovascular disease caused or exacerbated by SHS — and who may still be exposed to SHS in multi-unit dwellings — may exercise their right to “reasonable accommodation”. For example, they might invoke the “nuisance clause” present in most leases, which prohibits residents or their guests from engaging in any activity that interferes with the peace or wellbeing of other residents.[20], [21]

### Strengths and limitations

The findings in this study are based on the trend analysis of a repeated, national and representative sample of U.S. adolescents, the NYTS. Its standardized questionnaire and sampling frame during 2000–2009 add to the study's strengths. However, this study is subject to a number of limitations. First, the wording of the questionnaire was markedly changed in the 2011 iteration of the survey, and thus we were unable to assess trends after 2009. Second, there is a possibility that recall bias may have resulted in an under-reporting of indoor SHS exposure. However, recall was limited to past seven days, a relatively short time, and not susceptible to high levels of bias. Third, these data apply only to youths who attend school and, therefore, are not representative of all persons in this age group. However, data from the Current Population Survey indicate that 98.5% of U.S. youths aged 10–13 years and 97.1% of those 14–17 years were enrolled in a traditional school in 2011.[22] Hence, our findings are generalizable to most adolescents in the U.S. Finally, due to the design of the questionnaire we were not able to identify specific sources of indoor SHS exposure or rule out other sources, such as patios.

## Conclusion

This study indicated a significant decrease in the proportion of adolescents who were exposed to SHS between 2000 and 2009, a period strongly linked to the adoption of smoke free policies within the U.S. Enhanced and sustained measures are needed to further reduce indoor SHS exposure in all indoor areas, including private dwelling areas. Regulatory policies specific to SHS, as well as population-based measures to reduce overall smoking prevalence and intensity may help lower prevalence and disparities in indoor SHS exposure among adolescents and youths in all indoor areas.
